# The 2020 Coronavirus Pandemic as a Change-Event in Sport Performers’ Careers: Conceptual and Applied Practice Considerations

**DOI:** 10.3389/fpsyg.2020.567966

**Published:** 2020-09-23

**Authors:** Roy David Samuel, Gershon Tenenbaum, Yair Galily

**Affiliations:** Interdisciplinary Center Herzliya, Herzliya, Israel

**Keywords:** athletes, coaches, referees, COVID-19, quarantine, detraining

## Abstract

The Coronavirus experience (CE) presents a highly challenging period for sport performers (e.g., athletes, coaches, referees), with potential effects on their lives and career trajectories. In this article, we initially conceptualize the CE using the scheme of change for sport psychology practice (Samuel and Tenenbaum, 2011a). Within this framework, the CE is understood as a longitudinal, multifaceted, unpredicted, non-controlled change-event, with four distinct stages: (a) a pre-Coronavirus stage with unique career contextual conditions (i.e., stable engagement or a transitional period), (b) Coronavirus stage-A accompanied by instability and confusion, emotional response, and cognitive appraisal, (c) Coronavirus stage-B characterized by active coping or regression, and (d) Coronavirus stage-C; instability endures or decreases, depending on career trajectory. The CE presents sport performers with modifications in various dimensions, including physical and physiological, motor skills, psycho-social and self-identity, relationships, performance and achievement, motivation and aspirations, organizational-occupational, and micro– and macro–cultural issues. Sport performers can exhibit several emotional responses (i.e., positive, negative, neutral), and consequential coping endeavors. The development of the change process is underlined by key decisions, manifested in sport performers’ attempts to implement responsive change in these dimensions (e.g., adapt their diets, sleep routines, and exercise regimen). The second part of the article discusses applied practice considerations, presenting various techniques and methodologies which practitioners can apply while consulting from a change-based perspective. Ethical issues pertaining to the formation of effective therapeutic relationships during this period are also assessed. The conclusions offer future avenues for researchers and practitioners when attempting to evaluate and cope with this global phenomenon.

## Introduction

The year 2020 was foreseen as a peak year for many sport performers (i.e., athletes, coaches, referees), during which the realization of dreams and abilities would be achieved. However, the emergence of the Coronavirus pandemic during February-March 2020 resulted in major global changes, including quarantine and lockdown, social distancing, and cessation of commercial flights. Naturally, these had a considerable effect on the world of sport, as athletes could not train or compete, as well as travel to international meets. Thus, most major sport events planned for 2020, such as the Tokyo Olympic Games (OGs), the Euro (European Championships in association football), and the Wimbledon tennis tournament, were either delayed or ultimately canceled (BBC Sport, 2020). Athletes were faced with considerable modifications of their lifestyle and routines, interpersonal relationships, financial status (e.g., a loss of a job or a sponsor), as well as with the loss of aspirations and self-fulfillment (Taku and Arai, 2020). Those who got infected by the Corona Virus Disease 2019 (COVID-19), experienced health concerns as well as insecurity related to physical, performance, and occupational status. Other sport performers, such as coaches and referees were also experiencing meaningful changes to their sport engagement and career trajectories, feeling less publicly essential than they were used to (Taku and Arai, 2020). It was suggested that elite referees faced issues, such as quarantine, time away from families and loved ones, a lack of ongoing contact with colleagues, and fiscal concerns (Webb, 2020).

In an attempt to evaluate the effects of the Coronavirus pandemic on Olympic/Paralympic athletes, the International Society of Sport Psychology (ISSP) published three editorials and commentaries. It was suggested that: “The postponement of the Olympic and Paralympic games represents a significant career disruption. It potentially involves a loss of identity, motivation and meaning” (Henriksen et al., 2020, p. 1). In another ISSP publication, this new situation was regarded as a “crisis transition,” with potential stress responses including decreased sleep and appetite, increased rumination, loneliness, and fear of the uncertain future and the alternation of the Olympic cycle (Schinke et al., 2020a). In a third publication, the influence of cultural context was considered as important in how professional athletes might respond to social distancing. Also, a centralized athletic identity might hinder psychological responses to social distancing during the quarantine period (Schinke et al., 2020b). Similarly, the Association for Applied Sport Psychology (AASP) suggested that during this period athletes might experience an emotional rollercoaster due to “the constant influx of information, changes to daily routines, uncertainty with personal health and the health of others coupled with rapidly changing reports.” This organization also published practical recommendations for athletes and practitioners to maintain mental health and effective support during this period (Byrd et al., 2020).

Pillay et al. (2020) circulated an online survey to elite and semi-elite South African athletes during the final phase (last week of April 2020) of their domestic level 5 lockdown. The results revealed that sleep patterns changed significantly during the quarantine period (79%; *p* < 0.01) and that a significant number of athletes consumed excessive amounts of carbohydrates (76%; *p* < 0.01). During this period, most athletes trained alone (61%; *p* < 0.01), daily (61%; *p* < 0.01) at moderate intensity (58%; *p* < 0.01) and for 30–60 min (72%). Furthermore, many athletes reported feeling depressed (52%) and have struggled to keep themselves motivated to exercise (55%). Concerning the return to competitive sport, 35% of the athletes expected to return within 1–3 months whilst 31% felt unsure. Only 50% of them were comfortable with returning upon authority approval, naming requirements, such as established protocols, risk mitigation strategies, and guidance/support from sports federations.

In a similar vein, di Fronso et al. (2020) have examined the perceived stress and the psychobiosocial states of competitive Italian athletes representing individual (e.g., tennis, golf, swimming; *n* = 539) and team (e.g., soccer, basketball, rugby; *n* = 593) sports, a month after the beginning of the Italian lockdown. The findings suggested that compared with the preceding period (i.e., data was generated from Italian norms of these factors), the COVID-19 crisis was associated with athletes’ higher levels of perceived stress and dysfunctional psychobiosocial states, and lower levels of functional psychobiosocial states. These effects were more dominant for women athletes. Moreover, Elite/expert athletes reported lower perceived stress and higher functional psychobiosocial states scores than novice performers, potentially reflecting their more attuned coping resources. As part of their coping efforts, most of the athletes (80%) reported being in contact via the web with their coaches or other professionals, emphasizing the importance of “staying connected.”

Likewise, Clemente-Suárez et al. (2020) surveyed 175 Olympic and Paralympic athletes from several sport disciplines, who had been confined in Spain at that time (March 24 to April 12, 2020). Academic and sport variables, individual perceptions about the COVID-19 crisis, and psychological factors were assessed. The findings indicated that Olympic and Paralympic athletes showed negative perceptions of the confinement with regards to their workouts, but not to their performance. Also, no significant impact of the quarantine in the anxiety responses of the athletes was evident. This could be attributed to the coping skills and experience of these high-performance athletes in coping with anxiety. Paralympic athletes felt a stronger impact of the confinement in their training and performance than Olympic athletes, suggesting that every athletic population might be unique in its response.

Reframing the Coronavirus experience (CE) within the various contexts of sport performers’ careers is imperative to enable the sport community to effectively respond and cope with this new and demanding situation. While many sport performers were negatively affected by this situation, others might have perceived it rather positively (Taku and Arai, 2020). For example, injured athletes had more time to recover and inexperienced Olympians had time to improve their skills (Henriksen et al., 2020; Schinke et al., 2020a). Thus, specific career contextual conditions can be seen to influence sport performers’ perceptions of and reactions to this unique situation. To allow effective support, it is vital to accurately define this experience, using valid conceptual frameworks. Thus, in this article, the CE is first evaluated using the scheme of change for sport psychology practice (SCSPP; Samuel and Tenenbaum, 2011a). Then, applied practice considerations are discussed. Finally, recommendations are suggested for future research.

## The SCSPP

The SCSPP (Samuel and Tenenbaum, 2011a) suggests that the athletic career is highly dynamic and is characterized by the appearance of various types of change-events that can disrupt the athletic engagement status quo, cause instability, and initiate a demand for change. Change-events can be characterized with a negative (e.g., *injuries*), a positive (e.g., *a transition to a higher level*), or a moderate (e.g., *modifications in sport regulations*) emotional profile, pertaining to perceived significance, perceived severity, emotional and cognitive reactions, perceived control, and effective/ineffective coping (Samuel and Tenenbaum, 2011b; Samuel et al., 2020a). The perceived significance of a change-event is related to athletic identity (AI; Brewer et al., 1993) as well as to the impact it creates in the athlete’s career (i.e., how much imbalance it creates to the career status quo). It is suggested that change-events which are “closer,” or more related, to athletic identity (e.g., injuries, transitions among professional levels) would be perceived by athletes as more significant and emotionally severe (i.e., either negatively or positively) in the context of the athletic career (Samuel and Tenenbaum, 2011a).

Upon experiencing a change-event in their careers, athletes engage in an appraisal process, in which they consider the characteristics of the event in the context of their careers, their existing coping resources, and potential solutions. Unique characteristics of change-events include: the perceived significance (i.e., how much change it creates in the athlete’s career), its temporal nature (i.e., whether it is a distinct event vs. a longitudinal process), whether it involves merely the athlete (e.g., an injury) or additional individuals (e.g., a conflict with a coach or a colleague), the dimensions in which the change occurs (i.e., whether it is mostly sport-related or includes various life dimensions), the timing in which the change occurs in athletes’ lives and careers, and the affective nature of the event (i.e., whether it is positive or negative; Samuel and Tenenbaum, 2011a).

Cognitively appraising the new situation, athletes then make a strategic decision as to how to initially respond to the change-event: (a) deny/ignore it, (b) cope independently, (c) consult with others, or (d) consult with a sport psychologist. Various factors might affect athletes’ strategic decisions, including: their perceived control over the situation, sport motivation (i.e., quality and intensity), athletic identity, existing coping skills, and available support (Samuel and Tenenbaum, 2011a). In deciding to avoid change, athletes typically remain in a state of emotional instability, unless the situation is favorably resolved without the need for coping or intervention. On the other hand, a decision to change means that the athlete is applying all necessary adjustments to effectively cope with the new situation. This conscious decision is moderated by the athlete’s motivation and capacity for change, the application of therapeutic processes (independently or professionally), and existing psychological support.

It is further assumed that motivated athletes who also maintain a capacity for change, and who receive adequate psychological support, will make the decision to change. Then athletes will attempt to implement the change in the relevant dimensions of their athletic engagement. In implementing the change, athletes will feel in control and will assume responsibility for initiating the change. As a result, they will tend to perceive the outcome of the change process more positively. However, a probabilistic perspective is adopted, recognizing that various factors can affect the outcome of the change process. The SCSPP has received empirical (e.g., Samuel and Tenenbaum, 2011b; Knowles and Lorimer, 2014) and applied practice support (Samuel, 2013). It was also expanded to examine the career change-events of additional sport performers, including coaches (Samuel et al., 2020a) and sport referees (Samuel et al., 2017; Samuel, 2019).

## CE as a Career Change-Event

**FIGURE 1:**
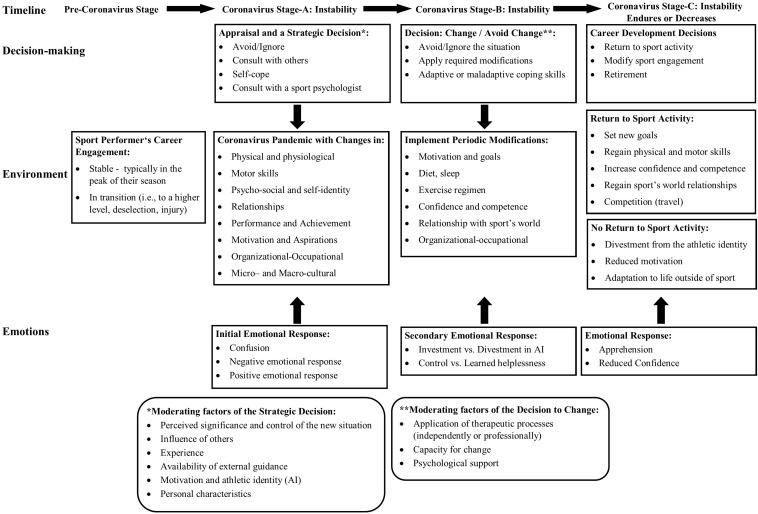
The Coronavirus experience as a change process in sport performers’ careers.

The conceptualization of the CE as a career change-event is presented in Figure 1. The development of the change process is presented along the time plane, the environment plane, the decision-making plane, and the emotional plane. Several career trajectories are consequently foreseen during and following the CE. Considering the unique characteristics of the CE and the changes it posed on sport performers’ careers, it could be classified as a longitudinal, multifaceted, unpredicted, non-controlled change-event. To a degree, we can compare the complexity of this unique experience to a severe injury or illness, which presents itself unexpectedly and forces sport performers to considerably change their sport engagement (see Samuel et al., 2015). However, dissimilar to an injury, which is an individual experience, the CE presents a shared-cultural experience; performers in comparable geographical locations were faced with the same challenges.

The sport performer typically experiences four stages of the change process: (a) a pre-Coronavirus stage with unique career contextual conditions (i.e., stable engagement or a transitional period), (b) Coronavirus stage-A accompanied by instability and confusion, emotional response (i.e., positive or negative), and cognitive appraisal, (c) Coronavirus stage-B characterized by active coping or regression, and (d) Coronavirus stage-C; instability endures or decreases, depending on career trajectory. In the pre-Coronavirus stage, sport performers were experiencing a stable sport engagement, typically being at the peak of their season or getting close to it (i.e., around February). At this stage, they had a specific career status (i.e., general sporting circumstances) pertaining to motivation, skills, and performance as well as personal lives, which could have affected their initial response to the Coronavirus emergence. Alternatively, performers could enter this new situation in an unstable and transitional status, affecting their readiness and capability to cope with the new situation. These included a transition to a higher professional level (e.g., cadets to junior, junior to senior, transition to a professional league, signing a new contract), contemplating of moving to play internationally, experiencing deselection from a club or national team, or approaching contract termination. They were required to manage these processes under much uncertainty, and making new decisions pertaining to their career development. For example, professional team-sport players who had contemplated moving to play abroad, now needed to decide whether to take professional and personal risks (e.g., travel internationally, financial uncertainty) and actualize this move in order to promote their careers, or alternatively maintain stability and remain playing in their domestic countries.

In the second stage, the Coronavirus had emerged, and sport performers were potentially experiencing career instability (Coronavirus stage-A, see Figure 1). They were perhaps required to reconsider the new situation and its potential impact on their careers. The initial emotional response might have typically involved confusion as there was much uncertainty. Athletes could have experienced changes in all facets of their engagement, as indicated in Figure 1. Depending on their evaluations of the new situation within the particular career context, certain athletes have arguably responded with a negative emotional response (i.e., maladaptive) while others have responded with a positive emotional response (i.e., adaptive). It is suggested that this stage also included active decision-making related to their initial response. In this stage, there were athletes who decided to disregard the new situation, cope with it independently, or consult with others. Among the latter, some also acknowledged the severity of this situation and obtained the advice of a sport psychology consultant. This strategic decision was moderated by various factors (e.g., perceived significance, motivation, experience), as indicated in Figure 1.

Following the initial perception of the new situation, athletes moved to the third stage, which arguably required them to maintain high motivation and invested athletic identity. While in many cases athletes did not have control over the environmental circumstances (e.g., whether their regular sport activity had returned or not), they still needed to assume control over their emotional response and their willingness to adapt their routines accordingly. Some athletes made a conscious decision to change, apply any required modifications to daily and weekly routines and lifestyle. For example, shotput coach, Dale Stevenson, commented on how adaptive athletes were during this period (World Athletics, 2020):

In the circumstances I’ve had to make a big shift away from control and to trust the athletes to self-direct… One athlete has improvised by using sandbags in training, although it is hard to know the weight of the sandbags. There are now great volatility and variations in terms of the training elements.

To effectively cope with the uncertainty, athletes could use adaptive coping strategies (Carver, 1997), including (a) instrumental support from coaches, fitness trainers, and nutritionists, (b) emotional support from family, friends, and consultants, (c) making action plans and taking direct actions to enhance their physical and psychological well-being, and (d) increasing their efforts be open to the new experience and vent their negative emotions out. Alternatively, athletes could decide to ignore the uncertain times and divest themselves from their sport engagement. The latter is well reflected in the following quote from tennis legend, Roger Federer (Khanna, 2020):

The truth is I’m not training because, to be honest, I don’t see a reason to do it right now. I’m physically fine and I think it will be a long time before we compete again. It’s important for me to take a good rest now. I don’t miss tennis so much, although when we are close to returning and I have a goal to train, I will be super motivated.

As indicated in Figure 1, during stages B and C, athletes probably experienced a range of changes in their sport engagement, requiring them to make psychological and behavioral adaptations. Considering the *physical and physiological dimension*, athletes and referees were forced to gradually or abruptly (i.e., depending on their global location and type of activity) reduce their training regimen, as they became more and more secluded. They could not exercise regularly, abandoned their competitive apparatus, and ignored their physical routines. Many athletes experienced “a detraining syndrome”; a clinical condition that might develop when athletes with a long endurance-training history suddenly abandon their regular physical activity (Andreato et al., 2020). This syndrome is characterized by a mixture of physical and psychological negative effects (Mujika and Padilla, 2000). For example, detraining had an impact on caloric balance and metabolism (i.e., increase in body fat and decrease in muscle mass), resulting in expected modifications to sleep and diet practices. Not being able to use their body as they were used to, athletes and referees might have felt restless, experiencing somatic anxiety and negative mood (Jukic et al., 2020).

Complementing the detraining effects, the longitudinal stress induced by the quarantine or lockdown, during stage-C, might have resulted in a feeling of *learned helplessness* (LH; Maier and Seligman, 2016), with ramifications for athletes’ physical and mental health. LH is a maladaptive motor and behavioral deficit in response to neural compromise. It was suggested that following an anterior cruciate ligament reconstruction, athletes are prone to experience LH as a result of being in a longitudinal inactive state, among other factors (Burland et al., 2019). To prevent the maladaptive syndromes athletes must have maintained in a high functioning neural and sensorimotor state during Coronavirus related quarantines/lockdowns. The fluctuations in stress and the modifications in exercise routines could result in athletic injuries or illness (Brink et al., 2010; Ivarsson et al., 2017).

In the *motor skills dimension*, the lack of engagement in physical activity could have led to reductions in speed, technical accuracy, sensorimotor coordination, and movement flow (Tran et al., 2017; Jukic et al., 2020). There is evidence that only 2–4 weeks of training cessation can cause a significant and marked loss of performance in both active populations and elite athletes (see Sousa et al., 2019). Athletes might feel that well-established movements (i.e., motor programs) are being lost as time passes. This feeling is especially relevant for those athletes who were at the peak of their season (e.g., attempting to qualify for the OGs). Similarly, referees might experience reductions in movement coordination and decision-making speed and accuracy (see Samuel et al., 2020). In addition, the uncertainty about coming back to training forms a high sense of frustration, as skills are diminishing continuously.

Injured athletes are a sub-population that could have experienced the CE in a somewhat conflicting manner. On one hand, this period allowed recuperation and the ability to resume fundamental skills as well as gain confidence in their physical readiness. Athletes could adhere to rehabilitation protocols more sensibly. On the other hand, those who refrained from medical support, might have experienced a highly negative period, which potentially slowed down their recovery. Loss of rehabilitation motivation could result in negative feelings and helplessness (Burland et al., 2019).

The *psych-social and self-identity dimensions* were also heavily influenced. Friesen and Orlick (2010) suggested that elite athletes may have extraordinary physical talents and capacities, but they are still “regular people.” While this contention may be important for consultants of elite athletes, in the minds of elite athletes they might not be “regular individuals.” Thus, having to face ordinary adversity pertaining to limited basic foods and supplies, physical and medical insecurity, social isolation, and financial insecurity, might have been very challenging for elite and professional athletes. They were no longer the center of attention as they were used to be, potentially experiencing a significant challenge to their AI.

Furthermore, to effectively cope with this new and uncontrollable condition, it is possible that athletes, coaches, and referees might have begun to distance themselves from the sport performer’s role. When asked by their surrounding how they were feeling, many responded: “I’m just fine”; yet, in fact, their lack of emotional turmoil reflected the divestment of identification with the athlete/coach/referee role. This process is experienced in athletes following a retirement (Lavallee et al., 1997) or deselection (Grove et al., 2004). In this context, Samuel et al. (2015) demonstrated the importance of maintaining a high AI during longitudinal rehabilitation from a severe injury. Therefore, athletes might feel a conflict between their desire to maintain a high AI and their athletic engagement being considerably modified. As suggested by Coulter et al. (2016), unlike traits, people choose their goals in light of the social contingencies afforded to them. They can be defined through their goals, values, and other characteristic adaptations. In the CE, sport performers compromise between conflicting values, for example, their wish to maintain recognition and their wish to maintain physical health at all costs. For example, Newcastle United football player, Danny Rose, commented (Skilbeck, 2020): “But just off the fact people are suggesting we should go back to football, it’s basically like we’re guinea pigs or lab rats, that we’re going to go back to this phase and see if it works or not.” Furthermore, athletes were required to adapt their self-narrative perspective to meet the new situation. Narrative identities give lives their unique and culturally anchored meanings (Coulter et al., 2016). For example, by accepting that they must train differently or that the financial situation of clubs has changed. In this context, coaches, referees, and supporting staff might have begun to feel as “non-essential” individuals and were required to modify this narrative to become useful and “continue to be a resource” for athletes, peers, and family (Byrd et al., 2020; Taku and Arai, 2020). Olympic track coach, Mersha Asrat, for example, believes his role during these challenging times is to put a plan in place to “survive the storm.” He commented that (World Athletics, 2020): “All my athletes need to be strong – a role model for others with their behavior… As a coach, I have to tell them that this will pass.”

In addition, sport performers were typically required to adapt to abrupt changes in *relationships with others*. Regularly, sport performers engage in many social interactions with the external world (e.g., fellow athletes, coaches, referees, support staff, fans, and media) and were now reduced to interactions with their close ones. Being confined to their homes, athletes were forced to adapt to a new role, “a family person,” which could have been overwhelming for some. Moreover, sport performers are used to engaging in a transnational lifestyle and now lost much of their sense of freedom. For example, The Brazilian horizontal jumps coach, Nelio Moura, found himself coaching in China when the Coronavirus emerged. Together with his athletes he had experienced the difficulties of international traveling and associated quarantine. He commented on this period: “with so much uncertainty regarding the outdoor season and beyond, the biggest challenge is to keep everybody motivated and positive about the future.” Furthermore, during this period he identified communication as a key. Therefore, Moura uses an Athlete Monitoring System to send training schedules and monitor training plans (World Athletics, 2020).

Athletes might also experience challenges associated with having a family member, friend, or close acquaintance become seriously ill or die due to COVID-19. The relevance of this issue may vary depending on the reach of this pandemic in a given athlete’s country or region. Athletes who experience COVID-19 related loss and grief might experience a range of physical, cognitive, emotional, and behavioral symptoms (see Anderson, 2010) as well as potential modifications in their identity and motivation, both in life and in the sport domain. In addition, as the family is often an important support resource in athletes’ careers, such a misfortune might significantly impair athletes’ ability to maintain their regular activity. For example, mixed martial arts fighter, Khabib Nurmagomedov, had lost his father and coach, Abdulmanap Nurmagomedov, on July 3, 2020, at the age of 57, after suffering a heart attack and pneumonia following a positive test for COVID-19. The athlete confessed the pain is still raw as he continues to grieve. He also revealed how challenging it has been for him to continue his athletic engagement (Jones, 2020):

We were like friends. He was my father and coach, we were always together. We were very close… Of course, I’m sad. If I tell you with a straight face that it doesn’t affect my training, that’d be a lie. It does affect me, I think about him all the time.

Considering the *performance and achievement dimensions*, in most cases, athletes and elite referees coped with an abrupt cessation of their seasons. For many of them, this occurred in March-April 2020, when a number of sports were in the midst of their competitive engagement (e.g., entering the playoffs, attempting to secure Olympic qualification). Not being able to satisfy their achievement motivation and perform was highly demanding. Initially, there was much confusion concerning what would happen with the world of sport. The governmental-organizational decisions concerning the delay of the Tokyo OGs and other major sport competitions, which developed over a few weeks, could be perceived as detrimental for many athletes who wished to actualize their current abilities. Thinking of maintaining their form for another year, or perhaps even required to requalify for the OGs, was challenging (Schinke et al., 2020a). However, for those who were not in their peak ability and for those injured athletes, this break could also be perceived as beneficial (Taku and Arai, 2020). Thus, for inexperienced Olympic athletes or those with “ability gaps,” gaining an additional year of preparation was perceived favorably (Henriksen et al., 2020; Schinke et al., 2020a). On the other hand, for athletes who expected to retire from their athletic engagement following the Tokyo Games or to begin a dual-career, the postponement was undesirable. For example, both Tom Ransley, a double Olympic medalist on the British rowing team, and Eddie Dawkins, the Rio Olympic track cycling silver medalist, had announced their retirement (Taku and Arai, 2020).

Naturally, performance and achievement modifications as well as parallel change processes had posed a great challenge to the athletes’ *motivation and future aspirations*. They were required to reevaluate the relationships with their sport engagement and reposition the role of sport in their lives. The uncertainty concerning the future combined with the absence of achievement goals could typically result in reduced effort to exercise and maintain physical ability during the quarantine (Pillay et al., 2020). This effect was intensified as most athletes were isolated from teammates and coaches (Byrd et al., 2020; Henriksen et al., 2020).

Considering the *organizational dimension*, athletes, coaches, and referees experienced much uncertainty and instability. The international and domestic governing bodies, as well as the sport clubs, entered into an unspecified period of financial insecurity and debated or decided to reduce the salaries of athletes, coaches, and referees. For example, the NBA announced a 25% reduction in players’ salaries (NBA, 2020). American college football coaches were also asked to make financial concessions (Sallee and Kercheval, 2020). In many cases, this resulted in mistrust and further affected the athletes’ and coaches’ motivation and confidence. Vealey and Vernau (2010) had emphasized the imperative role of “organizational confidence” in building athletes’ self-confidence. In addition, there were several conflicts concerning the return to play protocols while considering quarantine and testing (MacInnes, 2020). Furthermore, in foresight, it seems that players and coaches would need to adapt to a changing international market, with fewer financial resources available worldwide leading to modifications in their market value, thereby creating alternated career trajectories (Transfer Market, 2020).

Athletes further experienced changes pertaining to the *micro- and macro-cultural dimensions*. In macro, they were exposed to global and national ramifications of the Coronavirus concerning quarantine and lockdown, health issues, death toll, and national morale. Some cultures maintain tighter family relationships and were heavily affected by the inability to meet with their extended family members (Schinke et al., 2020b). In micro, each nation and sport discipline reacted differently to the new situation and posed specific regulations. For example, while the French first league football season was canceled by the French government, in other nations there was a decision to continue or resume playing (Aarons and Lowe, 2020). Sport performers affiliated to sport disciplines that were canceled for the season might felt disappointed and even embarrassed by the organizations’ inability to resume activity. In addition, it is also possible that the meaning of values that society places on sports (i.e., societal values, such as “athletes are supposed to make a full commitment for training and win for the country”) have been challenged by the COVID-19 pandemic. This is especially eminent when an athlete’s personal values (e.g., “my priority is to enjoy each moment regardless of the consequences”) might contradict the societal ones (Taku and Arai, 2020).

Finally, athletes experienced modifications in the *technical-technological dimension.* During Coronavirus stage-B, they adapted to new technologies, such as Zoom-based workouts and other new communications. Archers, for example, were inclined to improvise home-made targets to maintain their skills. Coaches were required to present athletes (and referees) with new training methods, considering social distancing as well as their unique physical and mental conditions. For example, marathon coach, Patrick Sang, commented on the challenges involved in long-distance coaching (World Athletics, 2020):

Our coaching is very much one on one and the “coach’s eye” is an instrument we use to see how the individual athletes are responding to the workload at any given time. The activation of the coach’s eye in these circumstances is not possible. You are relying on the feedback of the athletes.

Therefore, each of them must have maintained adequate physical and mental abilities to cope efficiently as possible with the pandemic constraints. For those who lacked with the twenty-first century skills (e.g., technical-technological skills, information management and communication skills, creativity), the transformation to a technological world might have been overwhelming (Ritter et al., 2018).

The last stage of the change process (Coronavirus stage-C, see Figure 1) typically involves the athletes getting out of the quarantine/lockdown and returning to sport activity under domestic restrictions. This period is characterized by much apprehension and a need to regain confidence as active performers. Those who decided to return to active engagement are required to set new goals for this period, regain their physical and motor skills, increase general self-confidence as performers and specific skill related competence, and reestablish their relationships with their sport environment. When possible, athletes (and referees) would need to regain their competitive edge, initially by competing domestically and later by traveling internationally. The latter would remain challenging until the Coronavirus is globally eradicated. Those who got infected with COVID-19 might feel embarrassment and guilt for potentially affecting their colleagues (Cox, 2020). Older coaches might feel at risk for infection and experience a conflict between maintaining their careers and risking their health (Young, 2020). In addition, it is possible that sport performers would experience a circular process going back and forth between attempts to restart their competitive activity and going into quarantine or lockdown.

Furthermore, the challenge of quickly returning to sport activity, and at the same time adapting to the new demands of the environment (i.e., maintaining social distancing, apprehension of becoming infected with COVID-19) could also result in heightened stress response and risk of injury (Ivarsson et al., 2017; Andreato et al., 2020) or illness (Brink et al., 2010). Competing or refereeing in high frequency, as part of an intense effort to finish domestic leagues, might also lead to overwhelming physical and mental demands. For example, 14 cases of injuries were reported in German football Bundesliga and Bundesliga 2 over the first weekend of return to action (MARCA, 2020).

In addition, the need to compete in the absence of a crowd can be challenging for those motivated by such factors. For example, at the beginning of March 2020, NBA star, Lebron James provided the following quote (The Guardian, 2020):

We play games without the fans? Nah, that’s impossible… I ain’t playing if I ain’t got the fans in the crowd. That’s who I play for. I play for my teammates, and I play for the fans. That’s what it’s all about. So, if I show up to an arena and there ain’t no fans in there, I ain’t playing. They can do what they want to do.

Interestingly, James, as his fellow NBA players, continued to play the 2020 NBA season without the presence of a live crowd. This, perhaps, is an indication that sports participants had to adapt to this new reality. Therefore, in terms of the change process, it is predicted that sport performers who adopt this career trajectory would gradually feel less instability, as they return to the status quo.

## Applied Practice Considerations

Consisting of the conceptual tenets of the SCSPP, Samuel (2013) developed a six-phase consultation framework to support sport performers in change-events. The initial three phases involve *assessment*: (a) identifying the change-event experienced, (b) understanding the client’s perception of the change-event experienced, and (c) recognizing coping efforts/strategies and support resources. The final three phases involve *application*: (d) activating processes of change, (e) offering a decision to change, and (f) supporting the client’s attempts to implement this decision. This framework has been used with various athletic populations, including athletes (Samuel, 2013; Samuel et al., 2020b) and referees (Samuel, 2019).

At the emergence of the Coronavirus, the world of sport could not immediately evaluate all aspects of this change-event. Moreover, data or research findings concerning the psychological impact of such events on athletes were unavailable. Thus, practitioners must have considered how this event could modify sport performers’ lives and careers. Educating athletes and their affiliates about the range of changes they are potentially experiencing is initially recommended, as this is a new phenomenon that they encounter. Engaging in a dialogue with the athletes and their affiliates better allows practitioners to assess their perceptions of the change-event and provide more effective support. It must be considered that sport performers are not necessarily negatively affected.

Having a “point of reference” is vital in psychological consultation. The fact that practitioners and athletes cannot accurately define the situation is impeding. For example, some clients might view the situation as *a break* or *off-season period* while others might perceive it as *a catastrophe* to their engagement. Thus, to maintain the athletes’ motivation and AI, practitioners can address this condition as *an injury recovery period*. Typically, during the recovery phase of an injury, athletes assume perceptions of control and apply high efforts to actively cope with the injury while adapting their lifestyle and routines (Samuel et al., 2015). By conceptualizing the situation like an injury recovery phase, rather than a break, practitioners might keep sport performers within the world of sport. For those who would return to competitive activity after 2–3 months, this could prove to be vital.

As part of the *assessment phase* of the consultation process, it is also vital to evaluate the client’s *coping efforts* and *support resources*. Here, it begins to be evident which sport performers are relying on their environment for support and who is instigating a divestment from the sport’s role. Using short inventories, like the Brief COPE (Carver, 1997), practitioners can assess whether sport performers are using adaptive or maladaptive coping strategies. For example, using a follow-up procedure to capture their active plans can result in a purchase of home fitness equipment and in a modification of their practice routines (World Athletics, 2020). Such a procedure might also assist in detecting disengagement behaviors or even substance use.

The *application phase* of the consultation process focuses on reaching *a decision to change*, using the *processes of change*. The latter reflect “the covert and overt activities that people engage in to alter affect, thinking, behavior, or relationships related to a particular problem or more general patterns of living” (Prochaska and Norcross, 1994, p. 12). In the CE, the decision to change reflects the athletes’ willingness to stay close to their sport engagement despite the uncertainty, and maintaining high AI and motivation. Practitioners can apply *consciousness-raising* to make their clients aware of the new situation and suggest potential cures (Prochaska and Norcross, 1994). C*atharsis* allows clients to express their negative emotions, such as fear and anxiety, frustration, and insecurity (Prochaska and Norcross, 1994). This can be achieved by discussing past experiences when they have dealt with adversity. Furthermore, practitioners can promote *self-reevaluation* which involves assessing how feelings and thoughts about oneself with respect to a problem, and includes techniques, such as *value clarification, healthy role models*, and *corrective emotional experience* (Prochaska et al., 1994). For example, during April-May 2020, “The Last Dance” series was broadcasted on Netflix (co-produced with ESPN films), featuring Michael Jordan and the Chicago Bulls. Discussing with athletes the adversity and coping in these players’ careers can increase awareness of the athletes’ own coping efforts and allow them to be inspired to assume control over their careers. This would be an example of the use of a process of change termed *dramatic relief* (Prochaska and Norcross, 1994) in which the athlete observes emotional scenes in the environment, such as movies, and reacts with cathartic feelings (Samuel, 2013).

Engaging in *self-liberation*—the process in which individuals are becoming more aware of their needs, their barriers and alternatives, believe that they can create change, and commit to this belief—is also important for generating a healthy life routine. Likewise, they can apply *contingency management*; a process of change that refers to the modification of contingencies in the environment to create a behavioral change in a particular direction (Prochaska and Norcross, 1994). These changes are manifested in setting new goals for the quarantine period (e.g., maintain fitness and strength levels) and thereafter for the return to activity period (e.g., increase speed and technical skills). It is further manifested in modifying dietary and sleep routines to accommodate for detraining effects as well as engaging in a home-based exercise regimen. Here, the practitioner must work with clients to self-regulate their stress levels to ensure that physical demands will prevent illness or injury. It is advised to introduce clients to mindfulness interventions/applications which allow stress management and increase sleep quality during this period (Li et al., 2018).

Another recommendation is to assist athletes in maintaining sensorimotor activation of their nervous system. For example, using a 15-min daily Zoom-based program in which athletes (and referees) respond to auditory (e.g., the practitioner’s clap of hands) and visual (e.g., the practitioner’s fist opens and closes) cues produced by the practitioner. Also, athletes can build tactical plans related to their sports and practice imagery scripts. For example, a judoka may watch videos of their potential competition opponents, identifying their main techniques and tactical behavior. Then, s/he can relax, close their eyes and visualize a competition fight against this opponent.

Practitioners are advised to discuss the influence of the situation on the athletes’ identities (Henriksen et al., 2020). On one hand, maintaining a high AI may prove beneficial for their coping and return to the sport. On the other hand, a centralized sport identity may lead to social isolation and increased anxiety in case of an unexpected postponement of the return to activity (Schinke et al., 2020b). In this context, practitioners must try to assist in preventing major reductions in their self-confidence and sport competence. Furthermore, professional, high-profile, athletes might find themselves in an internal conflict whether to engage in their sport at all costs (e.g., risk of getting infected with COVID-19; reduced salaries) including competing with no crowd, or whether to postpone their return. In a sense, this situation (i.e., competing with no crowd) poses a demand to deprive esteem needs to fulfill self-actualization needs.

The decision to change is also manifested in maintaining working relationships with fellow athletes and not disengaging from such relationships. Relationships during quarantine might reduce in quality and attention is required to regain rapport. Finally, practitioners must facilitate athletes’ attempts to reorganize and define their relationships with their organizations and employers. This may involve signing new contracts and finding viable solutions to various problems. During this phase, the practitioner must be aware of the dynamic nature of the CE, which might develop in a non-linear manner. Thus, establishing a task-oriented mentality (Duda, 2005) using short-term goals can prove useful during the implementation phase. Self-regulation (Vealey and Vernau, 2010), through self-feedback sheets and coach’s feedback, is also imperative to increase skill competence and prevent an injury.

Athletes’ and their affiliates’ attempts to reach a decision to change are further moderated by their capacity for change and existing psychological support. The individual’s capacity for change is a multidimensional concept comprising of motivation for change, the expectancy of therapy, and coping styles (Samuel and Tenenbaum, 2011a). As indicated by Roger Federer’s quote, not all sport performers are motivated to respond to this new situation with an adaptive change. Also, practitioners are recommended to tailor the type of psychological support to suit their client’s coping style. *Internalizers* are self-critical, withdrawn, and self-reflective (Beutler et al., 2002). They would mostly benefit from insight-oriented therapy in which they discuss the CE in the context of their careers as well as their identity modifications. *Externalizers* are impulsive, action or task-oriented, and lacking insight (Beutler et al., 2002). They would mostly benefit from symptom-focused and skill-building therapy, involving goal-setting, imagery, and sensorimotor activity. In addition, it is important to assess the therapeutic alliance between the consultant and the client (Bordin, 1994). During the Coronavirus period, practitioners redefined their relationships with their clients, addressing issues, such as payments and methods of delivery (e.g., through online applications, such as Zoom). Clients must feel that they trust the guidance of consultants in such an unstable period of their careers. It is possible, that developing new therapeutic relationships during such a period and reaching “a bond” (i.e., the association of shared activity that is felt in terms of liking, trusting, and respect for each other, Bordin, 1994) can be highly risky and perhaps ineffective for practitioners.

From an ethical standpoint, it is not easy for practitioners to demand that sport performers act positively and that they can continue maintaining healthy sportive lifestyles. Without a clear future and sport-related goals, what gives practitioners the right to keep a positive mindset? Yet, considering the alternative, and the debilitative effects of detraining and learned helplessness on their clients’ physical and mental health, practitioners must assume a generally positive standpoint. In doing so, they must first assess their own perceptions of the situation and consider its effects on their lives. Many practitioners probably experience financial and occupational challenges; yet must continue serving as anchors in their clients’ lives, by modeling effective coping. Nevertheless, maintaining a caring and non-judgmental consulting perspective (Friesen and Orlick, 2010) during such a change process is also imperative, as each individual can perceive this event differently, with much influence to his or her current unique career and life situational context. Practitioners must acknowledge that creating an effective therapeutic setting using online applications, such as Zoom is challenging for both themselves and their clients. Certain clients might experience emotional as well as procedural (e.g., noise, unstable internet service) issues in therapeutic online sessions and should be supported (Van Bavel et al., 2020). As recently suggested by Van Bavel et al. (2020), the widespread term “social distancing,” implying that one needs to cut off meaningful interactions, is better replaced by “physical distancing” because it allows social connection even when people are physically separated. This issue must be discussed with athletes when practitioners are establishing non-physical therapeutic, web-based, therapeutic engagements.

## Conclusion and Future Recommendations

This article presents an initial conceptualization of athletes’ and other sport performers’ responses to the CE. The dynamic and longitudinal nature of this change-event suggests a highly complex and individualized experience. Many factors influence athletes and their affiliates in parallel, and thus the psychological response can be overwhelming. Therefore, much research is required to evaluate the influential factors as well as the athletes’ responses to this unique situation. A distinction must be made between the emotional response to the CE and the coping response. Athletes can react with negative emotions and still produce effective change processes as a result of active decisions and adaptive coping efforts. Alternatively, they might respond positively to the CE yet begin to divest from their sporting role.

The present global situation offers researchers a unique opportunity to concurrently evaluate the CE. From a career transition standpoint, this is methodologically invaluable. Still, as for most sport performers, the change process is not concluded; and so, it is also difficult to genuinely evaluate this experience within the context of their careers. Thus, in addition to concurrent longitudinal investigations, it is vital to conduct a future assessment of this type of change-event. Cross-national designs are also required, as the unique national and cultural contexts can affect the way this change-event is manifested in the sport career.

It is also worth evaluating the effectiveness of tele-consulting (Brueckner, 2019) during the CE, when sport performers are experiencing issues in various dimensions of their lives and sport engagement. Can practitioners and clients engage in meaningful and effective therapeutic counseling or rather focus on mental skills training? Finally, researchers and practitioners must acknowledge their own contexts as they have also been experiencing CE. This article was written by Israeli authors. The first author was fortunate to continue his teaching and applied practice with athletes, coaches, and soccer referees throughout the CE. The other two authors were engaged in teaching and research throughout this period. This has influenced our optimistic and positive perspective of this situation. We are, aware, however, that in each country, sport scientists are dealing with complex situations that would ultimately influence their attitudes toward the CE.

## Author Contributions

RS, GT, and YG wrote the lit review, study, and conclusions. All authors contributed to the article and approved the submitted version.

## Conflict of Interest

The authors declare that the research was conducted in the absence of any commercial or financial relationships that could be construed as a potential conflict of interest.
